# HCC-Derived Exosomes: Critical Player and Target for Cancer Immune Escape

**DOI:** 10.3390/cells8060558

**Published:** 2019-06-08

**Authors:** Qiuju Han, Huajun Zhao, Yu Jiang, Chunlai Yin, Jian Zhang

**Affiliations:** Institute of Immunopharmaceutical Sciences, School of Pharmaceutical Sciences, Shandong University, 44 Wenhua West Road, Jinan 250012, China; hanqiuju@sdu.edu.cn (Q.H.); huajunzhao08@126.com (H.Z.); jiangjiang53@163.com (Y.J.); yinchunlai1990@163.com (C.Y.)

**Keywords:** HCC, exosome, immune escape, miRNA

## Abstract

Hepatocellular carcinoma (HCC) is a primary malignancy of the liver, and currently the second most common cause of cancer-related deaths worldwide with increasing incidence and poor prognosis. Exosomes are now considered as important mediators of host anti-tumor immune response as well as tumor cell immune escape. HCC-derived exosomes have been shown to attenuate the cytotoxicity of T-cells and NK cells, and promote the immuno-suppressive M2 macrophages, N2 neutrophils, and Bregs. These exosomes harbor several immune-related non-coding RNAs and proteins that drive immune-escape and tumor progression, and thus may serve as potential diagnostic biomarkers and therapeutic targets for HCC. In a previous study, we identified miR146a as an exosomal factor that promotes M2-polarization and suppresses the anti-HCC function of T-cells. In this review, we summarized the role of tumor-derived exosomes and their key components in mediating tumor immune escape during HCC development.

## 1. Introduction

Hepatocellular carcinoma (HCC) is now the second most common cause of cancer-related deaths worldwide with a high mortality rate, and shows a low response rate to clinical interventions. The inherent immuno-tolerant characteristics of the normal liver translates to poor immunogenicity of HCC cells and an immunosuppressive tumor microenvironment, which limits the possibility of immuno-therapeutics. HCC cells remodel the tumor microenvironment through various mechanisms that enable them to escape immune surveillance, ultimately promoting tumor proliferation and metastasis. The HCC cells can induce immune cell death via the FasL/Fas and PD-L1/PD-1 pathways, resulting in a decrease in the number of T-cells and NK cells. In addition, they also recruit the immuno-suppressive Tregs and myeloid-derived suppressor cells (MDSCs) that inhibit CD8+ T-cells, resulting in tumor immune escape [1]. 

**Figure 1:**
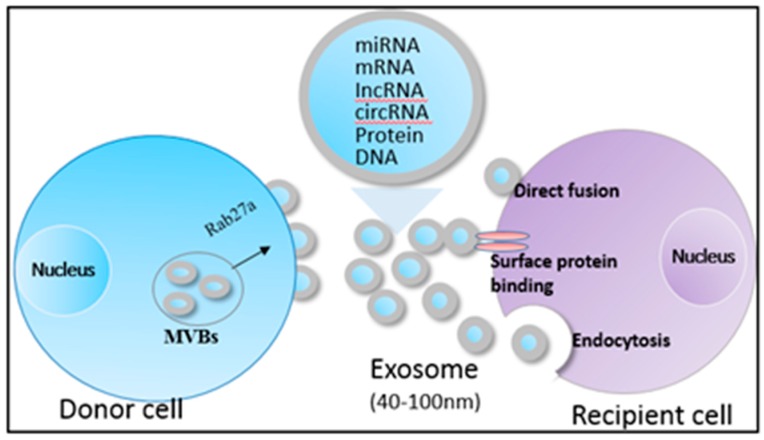
Biogenesis and contents of HCC-exosomes. Exosomes harbor proteins, mRNAs, miRNAs, lncRNAs, circRNAs, and DNAs, and transfer them to the recipient cells via direct fusion, binding with surface proteins and endocytosis.

Recent studies have shown that exosomes have a potential to regulate anti-tumor immune responses. Exosomes are nano-sized (40–100 nm) membrane-bound vesicles that are secreted by almost all cell types under both normal and pathological conditions. They are usually detected in biological fluids like blood, urine, and ascitic fluid. Exosomes transport various biomolecules, such as proteins, messenger RNAs (mRNAs), microRNAs (miRNAs), and long non-coding RNAs (lncRNAs) (Figure 1) [2,3]; common exosomal markers include HSp70, CD9, CD63, and CD81 [4,5]. The release of exosomes is a complex multi-step process, and neutral sphingomyelinase 2 (nSMase2), phosphorylated synaptosome-associated protein 23 (SNAP23) and Ras-related RAB proteins (RAB27A/RAB27B) are demonstrated to regulate exosome secretion from several cancer cells like HCC, melanoma, and colorectal cancer [6,7,8].

Although exosomes have been studied for several years, their biological significance is just beginning to be understood in cancer. The RNAs and proteins in the HCC-derived exosomes are different from those in the exosomes derived from normal hepatocytes. Studies show that exosomes mediate inter-cellular communication, between similar as well as different cell types. In the context of HCC, exosomes derived from Hep3B-cells carry functional mRNAs and miRNAs, and could be taken up by HepG2 cells [9]. Importantly, exosomes from HCC can remodel the tumor immune-environment through different ways, modulating anti-HCC immune responses [9]. Therefore, exosomal components are potential diagnostic and therapeutic biomarkers of HCC. 

## 2. Characteristics of HCC-Derived Exosomes

Transcriptomic analyses of HCC-derived exosomes indicate an abundance of RNAs of lengths ranging between 500–4000 bp—suggesting mRNAs and lncRNAs—with negligible amounts of ribosomal RNAs (18S and 28S rRNA) compared to their parental cells e.g., HKCI-C3, HKCI-8, and MHCC97L cell lines [10]. Interestingly, the HCC exosomal mRNAs can be translated into proteins in the recipient cells [10,11]. Furthermore, some small RNAs have also been detected in exosomes from HCC cell lines and HCC-derived primary cells [10,12]. Yu et al. found that miRNAs accounted for 3% of the small RNA repertoire in the exosomes of HCC patient-derived cells (PDCs), and their lengths differed from that in the donor cells. Due to variations in isolation methods, miRNAs account for 2–7% of all small exosomal RNAs obtained from supernatants of HCC cells cultured in vitro [13]. A total of 134 miRNAs were identified in Hep3B-derived exosomes, 11 of which (e.g., miR-584 and miR-517c) were only expressed in the exosomes and not the donor cells [9].

Mass spectrometry analysis has also identified 213 proteins in HCC-derived exosomes, of which 158 are overexpressed in exosomes derived from highly malignant HCC cells. Most of these proteins are exosomal markers and exosome secreting-related proteins, such as structural proteins, heat shock proteins (HSPs), syndecan-syntenin-ALIX, Ras-related proteins (RRAS), and vacuolar protein sorting-associated proteins. RAB27A/B, CD44, CDC42, and CLND3 are among the HCC exosomal proteins that are involved in carcinogenesis and metastasis [10], while the S100 calcium binding protein A4 (S100A4), caveolin-1 (CAV1), and CAV2 are enriched in metastatic HCC-derived exosomes both in mRNA and protein forms [10,11]. In addition, the exosomal membrane proteins are associated with their internalization by recipient cells [14].

## 3. HCC-Derived Exosomes are Critical for Immune Escape

### 3.1. Monocytes or Macrophages

Monocytes are non-terminally differentiated precursors of macrophages, and their fate is regulated by various stimuli. It is reported that HepG2 cells-derived exosomes can deliver phosphorylated receptor tyrosine kinases (RTKs) to monocytes through membrane fusion, leading to the activation of the downstream MAPK (Ras-Raf-MEK-ERK) signaling pathway, which blocks apoptosis by preventing caspase cleavage [15]. This might be critical for the accumulation of tumor-associated macrophages (TAMs) in the tumor microenvironment, but the exact exosome-associated functional molecules need to be identified in order to further understand the mechanism of immune escape involving monocytes.

Macrophages are abundant in the liver and critical participants of the innate immune response, and are increasingly being considered essential in the HCC microenvironment. In response to tumor-derived stimuli, macrophages can be polarized into the classical (M1) or alternative (M2) phenotypes. While the M1 macrophages display anti-tumor activity, the M2 macrophages are pro-tumorigenic. The TAMs switch from the M1 to M2 phenotype during HCC progression from the early stage to a more advanced stage [16,17]. The exosomes derived from HCC cells promote macrophage activation and M2 polarization through several mechanisms [18,19,20,21], and enable the tumors to escape immune surveillance.

LncRNA TUC339 is highly expressed in the HCC-derived exosomes that are transferred across HCC cells to promote tumor growth and metastasis [18]. Furthermore, exosomal lncRNA TUC339 could be transferred to neighboring macrophages to regulate the M1/M2 polarization and dampen the anti-tumor immune response in vitro [19]. Microarray studies revealed that the Toll-like receptor (TLR) signaling and FcγR-mediated phagocytosis pathways are downregulated in the macrophages by exosomal TUC339, and TUC339 knockdown increases their phagocytic activity. TUC339 is also involved in cytokine and chemokine receptor signaling pathways [18], although the exact mechanism needs further elucidation.

Tumor cell-derived exosomes also carry different miRNAs to the macrophages that regulate the expression of immune response-related genes. miR150 is highly expressed in the plasma of HCC patients as well in HCC-derived exosomes, and promotes the secretion of vascular endothelial growth factor (VEGF) from TAMs. Tumor-bearing mice treated with the miR150 inhibitor showed lower VEGF levels in the plasma and tumor tissues [20]. The HCC exosomal miR-23a-3p upregulates PD-L1 expression in macrophages via the STAT3 signaling pathway, which attenuates the anti-HCC immune response in vitro and in vivo [22]. Interestingly, exosomes derived from melatonin-treated HCC cells reverse the immunosuppressive status, demonstrating by downregulation of PD-L1 expression on macrophages in vitro and in vivo [21]. In a previous study, we observed that the proportion of CD11b^+^F4/80^+^CD206^+^ macrophages was significantly increased following exposure to HCC-derived exosomes, and accompanied by the upregulation of M2-specific markers including ccl17, ccl22, and arg-1. The M2 polarization both in vitro and in HCC-bearing mouse model was driven by the exosomal miR146a, which was directly regulated by the zinc finger transcription factor SALL4 in HCC cells [23].

### 3.2. Neutrophils

Neutrophils are key players of the innate immune system, although their role in mediating anti-tumor immune response has been largely ignored due to their short life span. Recently, neutrophils have emerged as a new kind of tumor-infiltrating myeloid cells, playing an important role in tumor growth and progression. Similarly to their myeloid ‘cousins’ macrophages, tumor-associated neutrophils (TANs) exert pro- as well as anti-tumor effects depending on their phenotypes in the tumor microenvironment [24]. While TANs can mediate tumor cell killing by releasing reactive oxygen species (ROS) and neutrophil elastase, they can also support early tumorigenesis by inducing angiogenesis through matrix metalloproteases 9 (MMP-9) and VEGF, and accelerate tumor proliferation by delivering neutrophil elastase [25].

Recent studies have indicated that tumor-derived exosomes increase the number of tumor-infiltrating CD66b+ neutrophils, and promote pro-tumorigenic N2 polarization in HCC, lung, gastric, colon, and breast cancers. Blocking the exosomal protein Rab27a decreased exosome secretion, and inhibited primary tumor growth by decreasing neutrophil recruitment [26,27]. Furthermore, exosomal tri-phosphate RNAs and high mobility group box-1 protein 1 (HMGB1) promote neutrophil recruitment and survival in the tumors by activating the NF-κB signaling pathway [26,28]. Recently, Yang et al. found that TGF-β was highly expressed in HCC-exosomes [29], which along with Axl-induced CXCL5, mediated neutrophil infiltration in HCC tissues and promoted tumor progression in HCC-models [30]. We recently found that the phenotype and function of neutrophils are regulated by HCC-derived exosomes, and the exact components of these exosomes need to be identified (unpublished data).

### 3.3. Dendritic Cells (DCs)

DCs play an important role in initiating both innate and adaptive immune responses. However, the tumor microenvironment disrupts DC maturation and activation, resulting in the formation of DCs with immunosuppressive potential, as seen in breast cancer and HCC [31].

The exosome-mediated interaction between cancer cells and DCs have highly divergent outcomes vis-a-vis anti-tumor immunity. Studies show that tumor-derived exosomes mediate immunosuppression by impairing DC differentiation and maturation via the IL-6-STAT3 signaling pathway [5], and by blocking the differentiation of myeloid precursor cells into CD11c+ DCs and inducing apoptosis, which decreases T-cell activity [32]. In contrast, exosomes containing tumor antigen might promote antigen presentation via the antigen-presenting cells (APCs) and activate the anti-tumor immune response [33]. For example, Rao et al. demonstrated that exosomes derived from the murine HCC cell line Hepa1-6 promoted DC maturation and activation, resulting in elevated levels of CD11c and the MHC classes I and II. In addition, the expression of costimulatory factors CD80 and CD86, and intercellular adhesion molecule were upregulated on DCs exposed to tumor exosomes compared to the untreated DCs, indicating that HCC-derived exosomes can activate DCs and increase the number of CD8+ T-cells. Although tumor growth was significantly inhibited by these tumor exosome-treated DCs in vivo, complete tumor eradication was not achieved [34]. It is possible that HCC exosomes also carry HCC-specific antigens that can trigger a strong DC-mediated immune response, and make the tumor microenvironment more conducive to the host immune cells.

Despite the stimulatory effect of HCC-derived exosomes on DCs, the immunosuppressive nature of the latter in the local tumor microenvironment in HCC patients should not be neglected [35], since it may not be accurately simulated in the orthotopic HCC transplantation models. In addition, exosomes have a highly complex molecular composition which also varies with HCC progression. It is therefore possible that DC-inhibiting factors were present at low levels at the time-points analyzed in these studies [34]. Furthermore, the preparation methods of exosomes may also influence DC regulation and function.

### 3.4. Natural Killer (NK) Cells

NK cells play an important role in anti-tumor response on account of their ability to distinguish between ‘self’ and ‘non-self’ antigens mainly by surface stimulatory and inhibitory receptors, which respectively transmit activation signals and inhibition signals. NK cell activation is determined by the balance between these signals [36]. However, tumor cells can induce NK cell dysfunction through various mechanisms, and escape NK cell monitoring. Exosomes from different tumor cells, including HCC, can be taken up by NK cells [37,38], although the underlying mechanisms are unknown.

The role of tumor-derived exosomes on NK cell function is intriguing. One study found that stress-induced HSPs, which are also transported by exosomes, act as endogenous “danger signals” that can increase tumor immunogenicity and promote NK cell activity [39]. A subsequent study found that HCC cells (HepG2 and PLC/PRF/5) cultured with ineffective anti-cancer drugs (e.g., irinotecan hydrochloride and carboplatin) released high amounts of HSP-carrying exosomes. Although the activating receptors CD69, NKG2D, and NKp44 were downregulated and the inhibitory receptor CD94 was upregulated on the NK cells, their cytotoxic activity in response to these exosomes was augmented due to granzyme B release [40]. Similarly, HSP70-positive exosomes derived from human melanoma cells augmented the cytotoxic response of NK cells, leading to diminished tumor growth [39].

NKG2D is a surface stimulatory receptor of NK cells, it interacts with ligands including the highly polymorphic MHC class I-related chains A and B (MICA/B) and the unique long 16 binding proteins (ULBP1-6). Stress-induced upregulation of NKG2D ligands (NKG2DLs) alone is sufficient to initiate NK cell activation and degranulation [41]. Soluble NKG2DLs (sNKG2DLs), such as MICA that is shed from the surface of prostate cancer, ovarian cancer, and leukemia cells, bind to NKG2D on NK cells, this blocks the recognition of NKG2DLs expressed in cancer, resulting in tumor immune escape from NK cell surveillance [4,42,43]. Previous studies reported that exosomal MICA inhibited NK cell cytotoxicity in several cancer types such as melanoma and leukemia. Consistent with this, elevated levels of soluble MICA have also been detected in the sera of HCC patients [41], and both MICA and MICB have been identified in the HCC-derived exosomes [44]. This indicates that MICA-harboring exosomes secreted by HCC cells likely inhibit NK cell function through competitive inhibition of agonistic NKG2D signaling. The possible involvement of other factors like ULBPs in exosome-mediated NK cell dysfunction remain to be studied.

In a previous study, we found that exosomes derived from CHB patients transmitted HBV to uninfected hepatoma cells. In addition, these exosomes were also able to transfer HBV components into human primary NK cells, and decreased their cytotoxic activity by dampening RIG-I expression and the downstream signaling pathways [45]. Since HBV infection is a major direct factor promoting HCC development and progression [46], impaired NK cell function due to persistent HBV infection may contribute to the progression of HCC [47]. Therefore, NK cell dysfunction by exosomes carrying HBV or HCV components could be the underlying mechanism of chronic infection and HCC initiation.

### 3.5. T-Cells

Tumor cell-derived exosomes can downregulate the TCR receptor complex CD3ξ and JAK3 in T-cells, thereby affecting the release of IL-2, IL-7, and IL-15, as well as T-cell maintenance and proliferation [48]. In addition, exosomes influence the CD4+ and CD8+ T-cell subsets through various mechanisms.

#### 3.5.1. CD4+ T-Cells

CD4+ T-cells play a cardinal role in antibody production and release, and the activation and expansion of CD8+ T-cells involved in immune surveillance. A typical immune system-escaping strategy employed by tumor cells is inhibiting the Th1 response and recruiting CD4+ CD25+ regulatory T-cells (Tregs). The activation of Tregs is hypothesized to be one of the most important immune escape mechanisms employed by tumors.

Tumor-derived exosomes decrease T-cell proliferation and effector functions directly and/or indirectly by inhibiting DCs. The 14-3-3 protein zeta (14-3-3ζ) is highly expressed in HCC cells, and promotes the proliferation and epithelial–mesenchymal transition (EMT) of HCC cells. Wang et al. found that 14-3-3ζ protein and mRNA levels were increased significantly in tumor-infiltrating CD4+ T-cells compared to PBMCs, indicating that it could be transferred to T-cells from HCC cells. The 14-3-3ζ^high^ TILs express lower levels of inflammatory cytokines (e.g., IFN-γ, IL-12, and IL-2) and higher levels of anti-inflammatory cytokines (TGF-β, IL-10, and IL-4) in DEN-induced mouse model [49]. In addition, since tumor-derived exosomes have contrasting effects on DC function, the outcome of CD4+ T-cell response may differ depending on the model system.

Although exosomes can be internalized into Tregs, the possible mechanism of exosome-mediated Treg regulation is cell surface signaling rather than uptake [38]. Tumor-derived exosomes can deliver CD39, CD73, and adenosine into resting Tregs, resulting in their activation along with transcriptomic changes [50]. Furthermore, co-incubation of CD4+ T-cells with tumor exosomes increased the levels of critical immunological factors such as CTLA-4, TGF-β, IL-10, and COX-2 [38,50]. In addition, exosomes derived from HCC cells promoted the differentiation of naive T-cells from Teff (CD4+IFN-γ+) to Treg (CD4+Foxp3+) lineage in vitro and in DEN-induced mouse model [49], and 14-3-3ζ was identified as the key meditator. These data underscore the potential of exosome-exposed Tregs in promoting tumor progression and immune escape.

#### 3.5.2. CD8+ T-Cells

CD8+ T-cells can potentially recognize and kill tumor cells. However, a large fraction of the tumor-resident CD8+ T-cells have phenotypic changes, with high levels of programmed death-1 (PD-1) and Tim-3, and low levels of the immune co-receptors CD27 and CD28, losing their anti-tumor functions [51]. Tumors including HCC could convert CD8+ T-cells from cytotoxic effectors to inhibitory ones. Although studies indicate that exosomes cannot be taken up by CD8+ T-cells [38], they can directly regulate CD8+ T-cell functions by surface binding/fusion, or indirectly by regulating macrophages, Bregs, and Tregs [52].

Since CD8+ T-cells also express NKG2D, sNKG2DL or inhibitory molecules delivered by exosomes can potentially disrupt CD8+ T-cell function in the tumor environment. Exosome-delivered MICA and ULBP can reduce the cell-killing function of T-cells by inhibiting NKG2D signaling pathway, leading to tumor immune escape [4,53]. In addition, exosomes carrying TGF-β can downregulate NKG2D on CD8+ T-cells, and prevent their activation [52]. Recently, Wang et al. demonstrated that 14-3-3ζ can be partially transmitted from HCC cells to naive T-cells via exosomes. They found that T-cell exhaustion markers (PD-1, TIM-3, LAG3, and CTLA-4) were increased in CD8+ T-cells expressing high levels of 14-3-3ζ [49], indicating that 14-3-3ζ delivered by HCC-exosomes correlated significantly with an exhausted phenotype of T-cells. In addition, tumor cells express high levels of programmed death-ligand 1 (PD-L1), which interacts with the PD-1 receptor on T-cells and enables the cells to evade the immune surveillance. Recent studies indicate that cancer cells package PD-L1 into exosomes, exosomal PD-L1 mediates immune escape of tumor cells [54], and circulating exosomal PD-L1 levels can therefore be predictive of the clinical outcomes of anti-PD-1 therapy, ALIX was demonstrated to be required for incorporation of PD-L1 from membrane into exosomes [55].

**Figure 2:**
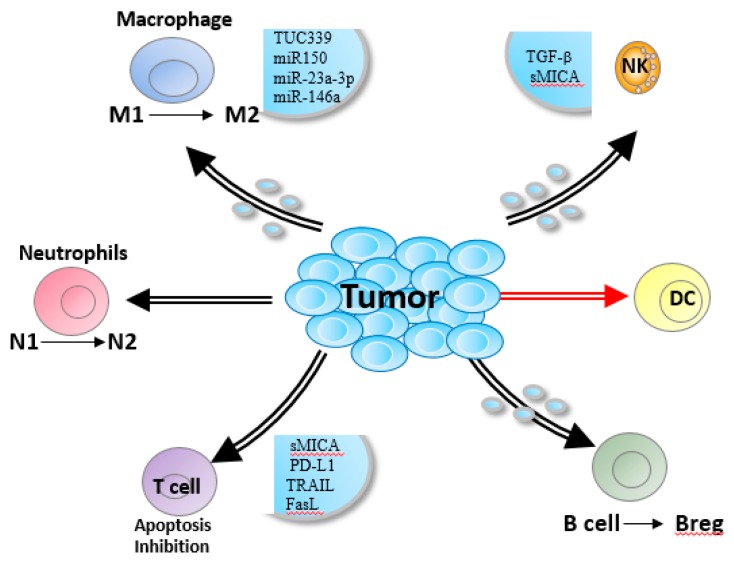
Roles of HCC-derived exosomes in regulating immune response. Exosomes suppress CD3 ζ-chain expression by T-cells and promoted their apoptosis by enriching for CD95L, TRAIL or galectin 9, and attenuate T-cell-mediated killing. Also, NK cell cytotoxicity is inhibited or blocked by TGF-β or soluble MICA/MICB in a NKG2D-dependent manner. Furthermore, exosomes can also promote the M2 macrophages, N2 neutrophils and Bregs.

HCC-derived exosomes also inhibit CD8+ T-cell function by promoting Bregs, Tregs, or M2 macrophages (Figure 2) [56]. Exosomes secreted from liver cancer cells increased PD-L1 expression on macrophages, which then decreased CD8+ T-cell frequency and IL-2 production, and increased T-cell apoptosis [22]. In a previous study, we found that HCC-derived exosomes promoted M2 polarization by the SALL4-miR146a axis. Furthermore, HCC-derived exosome-educated macrophages promoted T-cell exhaustion by upregulating PD-1, TIGIT, and CTLA4 on CD3+ T-cells, and accelerated HCC progression [23]. These findings are consistent with the role of miR146a in decreasing the number of tumor-infiltrating CD8+ T-cells in colorectal cancer [7].

### 3.6. B-Cells

B-cells are the central component of humoral immunity, and in addition to producing immunoglobulins (Ig), are also involved in antigen presentation. The regulatory B (Breg) cell subset producing high levels of IL-10 is known to accumulate in tumor environment. Breg suppresses the host immune responses and exerts a pro-tumorigenic effect in HCC, and is associated with advanced disease stage and poor prognosis [57].

Like monocytes, the activated, but not the resting, B-cells are also able to internalize tumor-derived exosomes [38]. Ye et al. recently identified a pro-tumorigenic TIM-1 + Breg subset exhibiting the immuno-phenotype of CD5^high^CD24^−^CD27^−/+^CD38^+/high^ in the context of HCC. Further study demonstrated that HCC-derived exosomes promoted expansion of this subset, resulting in strong suppressive activity against CD8+ T-cell via the HMGB1-TLR2/4-MAPK pathway, and providing favorable conditions for HCC progression. In addition, the infiltration of TIM-1 + Breg cells significantly correlated with disease stage and survival of HCC patients [56]. Xiao et al. identified another subtype of B-cells, which are highly expressed of PD-1, and this PD-1^hi^ B-cell subtype displayed a unique CD5^hi^CD24^−/+^CD27^hi/+^CD38^dim^ phenotype in advanced-stage HCC patients. Upon PD-1 activation, these cells produced large amounts of IL-10 that potently suppressed tumor-specific T-cell immunity [58], although the role of HCC-exosomes in this process needs clarification. Taken together, the immune-related miRNAs or proteins harbored in exosomes may promote the differentiation of immuno-suppressive B-cell subtypes in the HCC environment.

## 4. Conclusions and Outlook

Exosomes play multiple roles in the interaction between HCC and immune cells, and mediate tumor progression and immune escape [59]. In addition to what illustrated above (Figure 2), whether HCC-derived exosomes affect other immune subsets needs more investigation. A recent study showed that the effector function of NKG2D-expressing γδ T-cells is substantially impaired in HCC, which could be via the sNKG2DL transported by tumor-derived exosomes [60].

In addition, exosomes are also released by innate and adaptive immune cells. For example, Treg-derived exosomes are known to suppress Th1 cells in a Let-7d-dependent manner [61]. Therefore, the precise nature of exosome-mediated interactions between HCC and the other cells in the tumor environment needs further investigation. Our current knowledge of exosome physiology, release, transportation, internalization, and cargo delivery is also limited, and mechanisms underlying exosome interaction with and modification of recipient cells also require more comprehensive study. The role of exosomes in HCC development and progression can also be better understood in terms of cellular exosome production, and the selective enrichment of HCC-specific RNAs/proteins. Also, immune cell-type specificity of response should be justified. Importantly, to monitor the dynamic of exosome release, transportation and reception at the single-cell level as well as the resultant effects of specific exosomal components will help determine exosome-orchestrated the interaction between tumor and immune cells.

Exosomes are increasingly emerging as promising biomarkers for HCC diagnosis and prognosis, and more sophisticated methods have to be developed for isolating, analyzing, and tracking exosomes, in order to achieve clinical utilization.
